# Seasonal Activity, Spatial Distribution, and Physiological Limits of Subterranean Termites (*Reticulitermes* Species) in an East Texas Forest

**DOI:** 10.3390/insects12020086

**Published:** 2021-01-20

**Authors:** Mark Janowiecki, Edward L. Vargo

**Affiliations:** 1 Department of Entomology, Texas A&M University, College Station, TX 77843, USA; Ed.Vargo@tamu.edu; 2 New Orleans Mosquito, Termite and Rodent Control Board, New Orleans, LA 70122, USA

**Keywords:** termites, ecology, competition, foraging, climate

## Abstract

**Simple Summary:**

For species with similar resource requirements to inhabit the same location, they must somehow divide or share resources. In subterranean termites, multiple species often co-occur where they consume decomposing wood. How these species partition wood resources within an environment is poorly understood. We characterized the foraging activity of three species of *Reticulitermes* subterranean termites in a single site for 28 months to investigate the means by which resources are partitioned. We tested if species utilize resources under different climatic conditions by comparing the termite activity in wooden monitors to measurements of soil temperature and moisture. Generally, *Reticulitermes* termites foraged more during warmer, dryer months but each species responded differently to soil temperature and moisture. We found that *R. flavipes* was able to forage for longer durations and continued foraging during periods of high soil moisture, *R. hageni* increased foraging under higher soil moisture, and *R. virginicus* increased foraging under lower soil temperature. These results suggest that resources may be partitioned through differential foraging activity in response to different environmental conditions.

**Abstract:**

One of the major goals of ecology is to understand how co-habiting species partition limited resources. In the eastern U.S., at least three species of *Reticulitermes* subterranean termites often occur in sympatry; however, little is known about how these species divide food resources. In this study, we characterized the foraging activity of *Reticulitermes flavipes* (Kollar), *R. hageni* Banks, and *R. virginicus* (Banks) across seasons to assess the impact of environmental conditions on resource partitioning. A field site consisting of two grids of wooden monitors was sampled monthly for 28 months. Foraging activity in all three species was correlated with the interaction of temperature and moisture. This correlation was influenced by temperature and moisture approximately equally in *R. flavipes*, whereas temperature contributed more to the correlation in *R. hageni*, and moisture contributed more in *R. virginicus*. These differences caused each species to preferentially forage during specific environmental conditions: *R. flavipes* continued foraging after high moisture events, *R. hageni* increased foraging under higher soil moisture, and *R. virginicus* increased foraging under lower soil temperatures. We attempted to explain these patterns by the species’ physiological limits; however, we found no differences in upper lethal limit, desiccation, or submersion limits across species. These results add to the overall understanding of resource partitioning by emphasizing the ability of multiple species to utilize the same resource under different environmental conditions and raise questions regarding the physiological and/or behavioral mechanisms involved.

## 1. Introduction

How species with similar resource requirements are able to co-exist in the same habitat is a major question in community ecology. Co-existence can be achieved through minimizing niche overlap by reducing interspecific competition relative to intraspecific competition. Selection for reduced interspecific competition can lead to co-existence through resource partitioning [1] and can occur through morphological adaptations, differences in foraging behavior, or differential resource use under preferred environmental conditions [2,3]. For example, anole lizards in the Caribbean feed on similar prey but species behaviorally partition these resources by perching and feeding at different parts of a tree [4]. Similarly, many species of cichlids occur in the African Great Lakes but they have varying dentition and head shapes that enable them to utilize different resources [5].

Species competing for the same resources can utilize different competitive strategies [6]. Some species specialize on resource discovery and quickly exploit items before other species can find them. For example, sessile terrestrial plants coexist in the same area through differences in colonization ability which enables species to use resources at different times [7,8]. Conversely, other species specialize on resource domination where they are able to seize and maintain resources for a long duration [9]. This strategy of resource domination is more efficient in groups of mobile organisms, particularly in social insects that can quickly recruit colony members [10,11].

Subterranean termites are social insects that feed on cellulose, which potentially leads to a large overlap in shared resources and fierce competition. For example, across much of the eastern U.S., three species of *Reticulitermes*, *R. flavipes* (Kollar), *R. hageni* Banks, and *R. virginicus* (Banks), occur in close sympatry. These species all feed on decomposing wood, sometimes even in the same log [12]. How these species are able to co-exist feeding on the same resources in the same environment is unknown.

Studies employing ecological niche modeling on *Reticulitermes* species have found that the different species largely overlap in their environmental requirements, but each species prefers slightly different conditions. Comparisons of niche specialization of *R. flavipes* and *R. virginicus* found niche divergence is primarily driven by average temperature [13] and summer temperature [12]. While temperature was the most influential factor, average precipitation and precipitation seasonality also contributed to niche divergence in these species [12,13]. How these slight differences in environmental preferences might be involved in partitioning resource use in the same habitats is unknown, but differences in foraging activity have been found at a local scale. In a central Texas oak savannah site, Houseman et al. [14] found that *R. flavipes* forages more in the hot, dry months, whereas *R. hageni* is more active in cool, wet months, suggesting that *Reticulitermes* species may use the same resources at different times depending on local environmental conditions.

Different activity patterns among species in response to environmental conditions may reflect variation in their physiology [15,16,17] as reflected in their limits to survive extreme conditions such as temperature and moisture. Although temperature tolerances have not been compared between species of *Reticulitermes*, small differences (within 2 °C) have been found in the critical thermal maxima and minima when comparing *Reticulitermes* to a closely related genus *Coptotermes* [18,19]. This suggests that there may be variability in thermal limits that could better adapt a species to forage during certain temperatures. To test for differential ability to survive times of high soil moisture, Forschler and Henderson [20] compared the submersion tolerances of two species of *Reticulitermes* and found *R. flavipes* was able to withstand submersion approximately 1.5 times as long as *R. virginicus.* An enhanced ability to survive submersion longer may provide a competitive advantage after flooding events. Further studies comparing physiological limits in *Reticulitermes* may provide a better understanding of how these three species may be able to use the same resources under different environmental conditions.

Despite previous studies suggesting that environmental conditions are important in shaping species-specific foraging activity in some species of *Reticulitermes* [12,13,14], it is still not well understood how the three most widespread termites in the eastern U.S., *R. flavipes*, *R. hageni*, and *R. virginicus*, co-exist on the same food resources. Directly comparing the foraging activity of these species at a single site in relation to seasonal changes in temperature and moisture may begin to clarify how these species reduce interspecific competition by preferentially utilizing resources under specific environmental conditions. In this study, we tested if species-specific timing of foraging activity of *R. flavipes*, *R. hageni*, and *R. virginicus* enable these subterranean termites to partition resources and co-exist in the same area. We assessed the impact of soil temperature and moisture on foraging activity and determined whether underlying differences in heat, desiccation, or submersion tolerances of these species predict the observed effects of temperature and moisture. We hypothesized that these species are able to co-exist because they forage under different environmental conditions enabling them to partition resources in the same habitat. Further, we hypothesized that species-specific responses to environmental conditions may be related, at least in part, to differences in their physiological tolerances.

## 2. Materials and Methods

**Figure 1:**
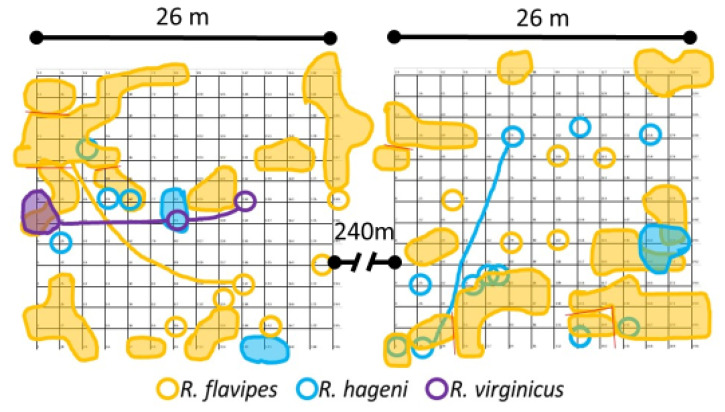
Cumulative locations and foraging areas of *Reticulitermes* colonies for the first six months of sampling. Samples were collected monthly. Shaded shapes indicate colony range, empty circles indicate single occurrence of colony, lines link samples distantly connected.

**Figure 2:**
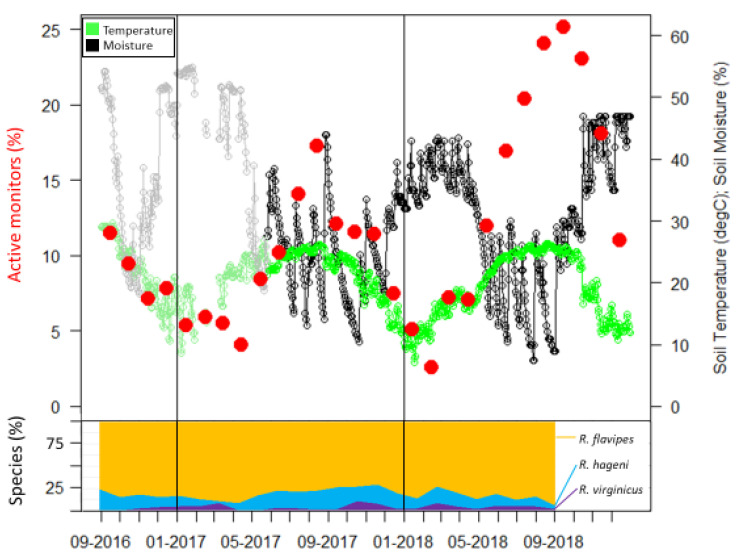
Active monitors, soil temperature, and soil moisture over 28 months. Species identifications were only determined for the first 24 months and percentages of each species are presented as a stacked line graph (bottom portion). The gray and light green portion of graph are data obtained from the USDA Natural Resources Conservation Service Soil Climate Analysis Network data for Beaumont, TX, USA.

Two 14 × 14 grids of wooden monitors spaced 2 m apart (Figure 1) were established at the Sam Houston State University Center for Biological Field Studies (30.744, −95.474) in eastern Texas between 26 and 29 July 2016. This site consists of a secondary growth forest in the Piney woods vegetational area dominated by large loblolly pines, *Pinus taeda* Linnaeus. The wooden monitors consisted of untreated loblolly pine stakes (3.8 cm × 3.8 cm × 61 cm) and were partially inserted 15 cm into the ground as in DeHeer and Vargo [21]. The two plots were separated by approximately 240 m. In each plot, four soil moisture sensors (S-SMD-M005, Onset Computer Corporation, Bourne, MA, USA) and one temperature sensor (S-TMB-M002, Onset Computer Corporation) were deployed on 26 May 2017. These recorded measurements every 2 min and were averaged for daily and monthly values. Data for the nine months prior to the use of the sensors were obtained from the United States Department of Agriculture Natural Resources Conservation Service Soil Climate Analysis Network data for Beaumont, TX, and are shown as the gray and light green portions in Figure 2.

Each wooden monitor was checked monthly for 28 months from September 2016 to December 2018. During monthly checks, each monitor was removed from the soil and externally examined for termites, minimizing disturbance of the monitor [21]. In a previous study in *Reticulitermes* spp., monthly sampling intervals were found not to impact termite foraging [22]. Each time we detected active termites, we placed four auxiliary monitors 1 m away from the original monitor in the four cardinal directions. This increased the density of monitors in more active areas [23], increasing the initial 392 monitors to 1834 monitors by the end of the study.

Samples were identified genetically to species through a combination of inter-simple sequence repeat (ISSR) polymorphisms [24] and 16S mtDNA sequencing [25] for the first 24 mo. Colony identity for each sample was determined using two highly polymorphic microsatellite markers, *Rf21-1* and *Rf24-2* [26] for the first six months, as this provided an adequate description of colony foraging patterns. Ten individuals per sample were genotyped when possible. Microsatellites were scored with the microsatellite plugin for Geneious v6.1.8 [27] and calculations used Genepop [28]. Samples from unique colonies had pairwise genotypic differentiation values that were significantly different (*p* < 0.05) from zero [29]. Colonies were unambiguously distinguished because of the high allelic diversity in the microsatellite markers (Table 1). Colony ranges were determined as the minimum area that encompassed all monitors a colony occupied. Voucher specimens are deposited in the Texas A&M University Insect Collection, College Station, TX, USA (#749)

Foraging duration was determined by the number of consecutive months that a monitor was active with the same species. Single month gaps, likely attributed to either foraging at low density or sampling error, i.e., not finding active termites, were filled, assuming foraging was constant. For example, a station that was active January, February, and April would be recorded as a duration of 4 months, assuming termites were also present, but undetected, in March. Foraging duration was calculated using custom Excel macros (v2016, Microsoft Corporation, Redmond, WA, USA). Discovery and leaving rates were calculated as in Chiu et al. [30]. The discovery rate was the total number of newly discovered monitors divided by the number of monitors not occupied that month, and the leaving rate was the total number of monitors left that month divided by the number of monitors occupied that month. The discovery rate was strongly correlated to the occupied monitors (*p* < 0.0001) because approximately 60% of the active monitors were newly active each month. Furthermore, the influence of temperature and precipitation on foraging was tested with a multiple linear regression analysis and the lmg (Lindeman, Merenda, & Gold [31]) relative importance metric was determined for predictors in the package “relaimpo” [32] within R v-3.4.1 [33].

The ability of each species to withstand temperature and moisture extremes was also assessed. To do so, the upper lethal limit (ULL) was determined by exposing individual termites (*n* = 16 individuals per colony) on a heat block (Thermo Scientific Compact Digital Dry Bath/Block Heater model 88871002). Two colonies of *R. flavipes*, *R. hageni*, and *R. virginicus* (*n* = 16 individuals per colony) were initially exposed to 33 °C and the temperature was increased 1 °C per 5 min. Termites were declared dead when no visible movement was detected. The previous temperature, the highest they survived, was recorded as its ULL. Colonies and species were compared with an ANOVA analysis in R v-3.4.1 [33] to determine differences in ULL.

To test the ability to withstand desiccation, groups of termites from two to three colonies of *R. flavipes*, *R. hageni*, and *R. virginicus* (*n* = 100 individuals per colony) were put into a desiccation chamber maintained at 0% relative humidity as done in Burdine and McCluney [34]. Mortality was visually assessed every 3–6 h until all individuals were deceased (54 h). Probit analysis in R v-3.4.1 [33] was used to determine the LT_50_ and LT_90_, or the time it takes to kill 50% and 90% of the individuals, respectively, to compare the ability to tolerate desiccation for each colony.

Submersion tolerance was assessed according to Forschler and Henderson [20]. Groups of termites (*n* = 10 individuals per time treatment, total of 70 individuals per colony) from two to three colonies of *R. flavipes*, *R. hageni*, and *R. virginicus* were forced underwater for variable amounts of time (1, 4, 8, 12, 16, 24, 36 h). When removed from the water, termites were placed on filter paper and mortality was assessed 24 h later. We determined the LT_50_ and LT_90_ for each colony in R v-3.4.1 [33] to compare the ability of each colony to survive submersion.

## 3. Results

Sampling occurrence was dominated by *R. flavipes*, followed by *R. hageni*, and then *R. virginicus* (Figure 2). In the first six months, there were 32 colonies of *R. flavipes*, 16 colonies of *R. hageni*, and a single colony of *R. virginicus*. One *R. hageni* colony in Plot 2 spanned a relatively long distance (more than 21 m) (Figure 1) and was confirmed with the addition of 11 microsatellite loci [26,35] in a species-specific multiplex [36]. Generally, foraging and monitor discovery increased with temperature and decreased with moisture for all three species (Figure 2). Colonies of the same species did not overlap, but colonies of different species overlapped, sometimes occupying the same monitor in consecutive months (Figure 1).

Although foraging activity was similar for the three study species, there were species-specific differences in the duration, monitor turnover, and impact of soil temperature and moisture on termite foraging. In the first 24 months, *R. flavipes* foraged longer (2.13 months) than *R. hageni* and *R. virginicus* (1.46 and 1.08 months, respectively) (F_2,1624_ = 31.14, *p* < 0.0001). During this time period, there were 165 total turnover events where one species took over a monitor occupied by a different species in the previous month (Table 2). Although *R. flavipes* had a disproportionate frequency of turnover events (*n* = 87), when scaled relative to the number of total occurrences, this species had relatively few turnover events (3.5%). In contrast, *R. virginicus* and *R. hageni* initiated foraging in a previously occupied monitor over a quarter and over an eighth of the time, respectively (Table 2). There were no intraspecific turnover events in colonies tracked during the first six months. The percent of occupied monitors and discovery rate were correlated to the interaction of temperature and moisture in a multiple regression for all three species (Table 3). Temperature and moisture made approximately equal contributions to the model for *R. flavipes*, whereas the occupied monitors and discovery rate of *R. hageni* was more influenced by temperature, and *R. virginicus* was more influenced by soil moisture (Table 3). The leaving rate was correlated to the interaction of temperature and moisture only in *R. hageni* and had an approximately even contribution of temperature and moisture (Table 3).

The leaving rate of *R. virginicus* was negatively correlated to soil moisture alone (*R*^2^ = 0.312, *p* = 0.02) but was not correlated to the combination of temperature and soil moisture. In *R. flavipes*, the leaving rate was not significantly correlated to temperature or moisture.

**Figure 3:**
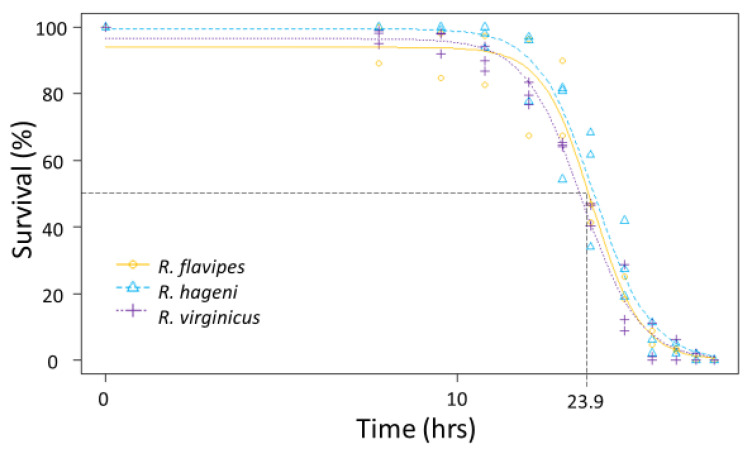
Desiccation tolerance for multiple colonies of *Reticulitermes flavipes*, *R. hageni*, and *R. virginicus*. Lethal time 50% (LT_50_), or the time at which 50% of individuals died from desiccation is indicated by the dashed line. The x axis is log-scaled.

**Figure 4:**
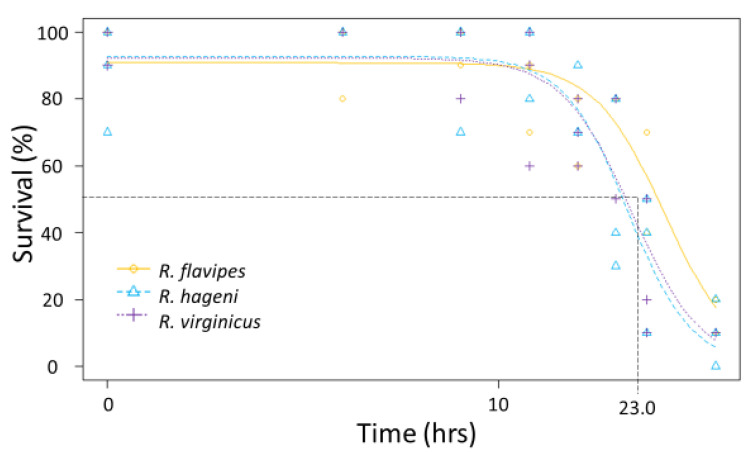
Submersion tolerance for multiple colonies of *Reticulitermes flavipes*, *R. hageni*, and *R. virginicus*. Lethal time 50% (LT_50_), or the time at which 50% of individuals died from submersion is indicated by the dashed line. The x axis is log-scaled.

Generally, there were no differences among species in the physiological limits examined. All three species had a similar ULL (average 42.6 °C, F_2,92_ = 0.642, *p* = 0.529). The LT_50_ for desiccation for the three *Reticulitermes* species was approximately 23.9 h; there was no significant difference among species as the colony variation within a species was as large as the variation between species (Figure 3) leading to overlapping 95% confidence intervals between colonies of different species. Similarly, the mean LT_50_ for submersion was 23.0 h, but there were no significant differences among species (Figure 4) because the 95% confidence intervals overlapped between colonies of different species.

## 4. Discussion

We found species-specific differences in foraging activity which broadens our understanding of how multiple *Reticulitermes* species can co-exist while minimizing interspecific competition. In our study, *R. flavipes* and *R. hageni* foraged more in months with high soil temperature, while *R. virginicus* foraging was less impacted by soil temperatures, thus allowing *R. virginicus* a competitive advantage during cooler periods (Table 3). As we observed that *R. virginicus* initiated foraging in monitors occupied by other species more than twice as often as the other two species, *R. virginicus* may utilize resources abandoned by the other species when the soil becomes cooler. Both *R. flavipes* and *R. virginicus* foraged more in months with lower soil moisture, while *R. hageni* was less impacted by soil moisture enabling it to forage more during times with higher soil moisture when the other two species were less active. Additionally, the leaving rate of *R. flavipes* was more resilient to environmental conditions which may provide this species a competitive advantage to continue foraging and not abandon food resources during times of extreme soil temperature or moisture levels that may cause *R. hageni* and *R. virginicus* to stop foraging. This was also evident from our observation that colonies of *R. flavipes* were more stable, occupying monitors for longer periods of time than colonies of the other two species.

Although our results show species-specific differences in foraging activity shaped by environmental conditions, we found no differences among species in heat, submersion, or desiccation limits. This is in contrast to Forschler and Henderson [20] who found differences in submersion tolerances between *R. flavipes* and *R. virginicus* (LT_90_ = 29.7 h and 23.0 h for *R. flavipes* and *R. virginicus*, respectively). However, this previous study involved only a single colony of each species and may not adequately represent colony-level differences within these species. Our results indicate that physiological limits may not be a good indicator of foraging activity in these species, potentially because of their ability to avoid extreme conditions through behavioral mechanisms. Subterranean termites forage in a three-dimensional network of tunnels and resources [14]. Thus, termites can move vertically through the soil profile to select preferred microhabitats. *Reticulitermes flavipes* has been observed to move 1–2 m below ground to avoid freezing temperatures in winter [37]. It is unknown whether different species tunnel at different depths according to species-specific preferences in temperature and moisture, but this could allow each species to choose preferred environmental conditions for tunneling further facilitating the partitioning of the foraging space.

In addition to differences in environmental conditions due to season and vertical stratification in the soil, microhabitat heterogeneity in the food resources themselves may play a role in resource partitioning. For example, the preference of *R. virginicus* to forage during cooler periods may indicate this species also feeds on cooler resources, such as old tree roots deep in the soil that the other two species are less likely to utilize. As *R. hageni* foraged more during wetter periods, this species may prefer resources with high moisture content, like food items in shaded, humid areas. For *R. flavipes*, we found the leaving rate was not negatively impacted by extreme environmental conditions, which may imply this species is better able to hold onto to occupied resources despite fluctuations in environmental conditions. In this way, the heterogeneity of microhabitats may reduce interspecific competition and facilitate the co-exist of *Reticulitermes* species. Further studies are needed to investigate the possible role of the microhabitat of food resources on wood utilization in subterranean termites.

The species-specific responses to different environmental conditions that we observed may allow each species to use the same resources at different times and these results are similar to previous findings in *Reticulitermes* [12,13,14]. Niche overlap has been studied by Maynard et al. [13] in *R. flavipes*, *R. virginicus*, and the invasive *Coptotermes formosanus* Shiraki, and by Hyseni and Garrick [12] in *R. flavipes*, *R. virginicus*, and the more regionally distributed *R. malletei*. When determining niche overlap in these species, both studies found air temperature most strongly separated the niches of *R. flavipes* and *R. virginicus*. We also found differences in temperature preference within our study site with *R. virginicus* foraging more during cooler soil temperatures compared to *R. flavipes*. When comparing the influence of temperature and moisture on foraging of *R. flavipes* and *R. hageni*, our results are similar to results found by Houseman et al. [14], who studied *R. flavipes* and *R. hageni* in central Texas. These authors found that foraging activity of *R. hageni* increased with higher soil moisture just as we found. However, these authors also found that soil temperature differentially influenced the foraging of *R. flavipes* and *R. hageni*, in contrast to our findings where there was no significant effect of temperature. This discrepancy may be the result of differences in the time intervals in which soil temperatures were measured since Houseman et al. [14] recorded temperature every 2 weeks whereas we measured temperature every 2 min, providing us with a finer scale view of the temperature foraging termites experienced. Overall, we generally observed similar foraging patterns reported in previous studies, but our results expand our knowledge by providing a more comprehensive view of the foraging activity in relation to environmental conditions of the three most widespread termites in the eastern U.S.

Although we observed resource partitioning of below ground foraging because our wooden monitors were inserted in the soil, our results have implications for partitioning above ground resources. Above ground wood debris is more affected by environmental conditions than below ground resources since it is more exposed to temperature and moisture without the buffering effect of soil. Different-sized wood debris would also be differentially affected by environmental conditions where larger wood debris would be expected to have smaller fluctuations in temperature and moisture, while small wood debris would likely have larger fluctuations. *Reticulitermes virginicus* is commonly found in larger logs [38] and *R. hageni* in smaller logs (pers. observ.). This difference aligns with our observations that *R. virginicus* foraging was more influenced by moisture, while *R. hageni* foraging was less influenced by moisture. By seeking out large, stable resources, *Reticulitermes virginicus* would be able to forage for a longer period of time buffered from moisture variation, while *R. hageni* is better able to withstand the variability encountered in smaller resources. However, this pattern does not hold for variation in soil temperature because we found foraging in *R. hageni* was more influenced by temperature than *R. virginicus*, even though *R. hageni* likely experiences more temperature variability in its preferred, small resource size. Clearly the role of moisture and temperature variation on above ground wood debris in determining resource use by different *Reticulitermes* species requires further research.

Extreme weather events can influence resource partitioning patterns by differentially influencing foraging activity. During our study, Hurricane Harvey brought approximately 50 cm of rain in a two-day period at the end of August 2017. This caused a steep decline in the percent of active monitors from August to September 2017 (Figure 2). Additionally, the winter of 2018 had a high level of precipitation, reflected by soil moisture levels similar to that of the hurricane (Figure 2). During both of these periods of high soil moisture, the number of active monitors sharply decreased due to a high overall leaving rate for those months, but the leaving rate differed among species. In our study the leaving rate was correlated to the interaction of temperature and moisture in *R. hageni*, and only to moisture levels for *R. virginicus*, but not to either temperature or moisture for *R. flavipes.* These results suggest that *R. flavipes* may be more adapted to survive flooding, which may provide it a competitive advantage after high precipitation events. Despite the apparent resilience of *R. flavipes* to high soil moisture content, this species was no more tolerant of submersion than were the other two species. As social insects, the ability to withstand extreme environmental conditions in termites may extend beyond the physiological ability of individuals and can be shaped by differences in colony level behaviors. For example, the fire ant *Solenopsis invicta* Buren, which originated in the Pantanal flood plain of South America, exhibits a behavior known as rafting in response to flooding where the entire colony forms a mass that contains the queen and brood and floats on the water until locating higher ground or the flood water recedes [39]. Although termites are unable to raft, differences in nesting habits may influence their ability to withstand floods. After Hurricane Katrina flooded parts of New Orleans, native *Reticulitermes* colonies stopped foraging in monitors but the invasive *C. formosanus* had only a minor decrease in foraging activity [40]. Although both genera are subterranean termites and nest in hollow cavities in wood debris, they have different nest structures; *Reticulitermes* builds a diffuse nest consisting of multicompartmental, mud and feces-lined chambers, while *Coptotermes* constructs a strong, carton nest built of soil and frass. In laboratory assays, groups of *C. formosanus* were able to withstand a longer duration of flooding when allowed to first create a carton nest [40] which may explain its ability to survive flooding longer than *Reticulitermes*. Although the three species of *Reticulitermes* in our study have nests of similar structure, differences in nesting location or nest permeability may account for the different response to soil moisture we observed.

In addition to resource partitioning, *Reticulitermes* termites may relocate their nest to prevent competition for disputed resources, or remain and directly compete. As *Reticulitermes* termites nest and forage in multiple wood resources connected by underground tunnels, they are able to relocate the reproductive center of the colony. It was long believed that *Reticulitermes* colonies were mobile and amoeba-like, constantly moving across an area [41]. However, our study, as well as a previous study [21], found colonies generally remain within a defined foraging area but switch between food resources within the forging area. When termites encounter competitors in their territory, they can relocate to a different resource or remain and fight. Although we did not observe direct interactions between termite colonies, we found a large number of turnover events (*n* = 165) where one species took over a monitor occupied in the previous month by a different species. This may be the result of competition between species but would require further investigation with a shorter inspection interval to determine whether colonies directly compete or simply move into an empty, previously occupied monitor. From these turnover events, our results suggest *R. virginicus* may be a better competitor since it took over occupied stations at a greater rate than *R. flavipes* and *R. hageni*. This may occur because *R. virginicus* generally has larger colonies with larger foraging ranges [42], enabling it to potentially overwhelm other species with its populous workforce. Additionally, *R. virginicus* may be more adapted to interspecific competition because we, and a previous study [21], observed few, widespread colonies of this species. This makes *R. virginicus* unlikely to encounter colonies of the same species and more likely to encounter colonies of a competing species. In contrast, *R. flavipes* and *R. hageni* are likely more adapted to intraspecific competition because their higher colony density increases the likelihood they will encounter opposing colonies of the same species. Understanding the competitive interactions between these species is an important area for future studies to determine the potential for species to partition resources through differences in competitive abilities.

While colonies may relocate their nest in response to competition, nest relocation may also occur in response to variable environmental conditions or in order to efficiently access higher valued resources. Species that are less adapted to endure extreme environmental conditions may be more likely to move among different resources in order to avoid exposure to harsh conditions. This movement between resources may cause species to forage over large areas in order to find and occupy food resources with similar microclimates. As *R. virginicus* generally forages over a more expansive area than *R. flavipes* and *R. hageni* [42], it might be expected to have more limited conditions it can tolerate since it may be compensating by inhabiting larger logs that are more dispersed throughout the environment. Despite the expectation that *R. virginicus* would be the least tolerant to variable environmental conditions, we found no differences in physiological limits among species. This suggests that other factors may influence the variation in the foraging range among these species. In addition to movement in response to environmental conditions, nest relocation may allow a colony to better utilize resources by moving the nest closer to high value resources in order to minimize resource transportation. This has been found previously in termites in response to the discovery of large food items [43,44,45] and ants in response to changes in prey availability [46,47]. However, future studies are needed to map both the foraging and nesting locations simultaneously to determine the factors that influence nest relocation.

Overall, we observed foraging patterns in *Reticulitermes* species and response to climatic factors similar to previous studies in different regions [12,13,14,21,44]. We found species-specific responses to environmental conditions; *R. flavipes* continued foraging after high moisture events, *R. hageni* increased foraging during high moisture, and *R. virginicus* increased foraging during low temperatures. However, further studies are needed, particularly for *R. virginicus*, because we included a small number of colonies (likely two, one from each plot) due to their expansive foraging areas. Nonetheless, these results contribute to the overall understanding of resource partitioning, showing that these species have unique behavioral responses to differences in soil temperature and moisture that allow them to co-exist while consuming the same resource. In our study, we assessed termite activity by adding wood monitors into an area to sample foraging colonies, enabling comparison between species because the wood resource was standardized for size and level of decomposition. However, termites may select resources that vary in size [38,44], tree species [48,49], or level of decomposition [50,51]. Future studies could investigate other factors that may influence how resources are partitioned in this group including the use of different food sources (e.g., wood species, size of wood debris, above versus below ground wood debris), the location of the nest relative to food sources, behavioral differences in the ability to defend food sources from opposing species, and microhabitat differences in temperature and moisture levels in the soil environment and inside above and below ground wood debris.

## Figures and Tables

**Table 1:** Microsatellite allelic diversity (average ± SEM) for *Reticulitermes flavipes*, *R. hageni*, and *R. virginicus* in southeast Texas at two loci.

Species (*n* = Number of colonies)	Allelic Diversity			
	*Rf21-1*		*Rf24-2*	
	Average Number of Alleles per Colony	Total Number of Alleles in Population	Average Number of Alleles per Colony	Total Number of Alleles in Population
*R. flavipes* (*n* = 32)	3.47 ± 0.67	41	1.81 ± 0.70	30
*R. hageni* (*n* = 16)	2.97 ± 1.06	18	2.44 ± 1.21	13
*R. virginicus* (*n* = 1)	4	4	4	4

**Table 2:** Species turnover in consecutive months for *Reticulitermes flavipes*, *R. hageni*, and *R. virginicus* from September 2016 to August 2018. Takeovers occur when a species inhabits a monitor that was active with a different species the previous month.

Takeover Species	Number of Takeover Events	Number of Takeovers Relative to Total Samples of That Species (%)
*R. flavipes*	87	3.54
*R. hageni*	51	12.56
*R. virginicus*	27	27.77

**Table 3:** Statistics of multiple linear regression for climatic factors (soil temperature and soil moisture) and foraging activity for *Reticulitermes flavipes*, *R. hageni*, and *R. virginicus*.

***R. flavipes***						
**Foraging Parameter**	**Multiple Regression Model**		**Partial Regression Components**			
	***R*^2^**	***p***	Variable *	**Reg. coef. ‡**	***R*^2^**	***p***
Occupied monitors	0.717	0.0005	Temp.	0.868	0.361	0.087
			Moist.	−0.525	0.356	0.095
Discovery rate	0.718	0.0005	Temp.	0.007	0.462	0.011
			Moist.	−0.003	0.256	0.578
Leaving rate	0.168	0.332	Temp.	−0.009	0.064	0.822
			Moist.	0.006	0.104	0.446
***R. hageni***						
**Foraging Parameter**	**Multiple Regression Model**		**Partial Regression Components**			
	***R*^2^**	***p***	Variable *	**Reg. coef. ‡**	***R*^2^**	***p***
Occupied monitors	0.437	0.0319	Temp.	0.124	0.337	0.039
			Moist.	−0.050	0.100	0.595
Discovery rate	0.560	0.0072	Temp.	0.001	0.432	0.012
			Moist.	−0.001	0.128	0.491
Leaving rate	0.576	0.0058	Temp.	−0.022	0.289	0.200
			Moist.	0.014	0.287	0.207
***R. virginicus***						
**Foraging Parameter**	**Multiple Regression Model**		**Partial Regression Components**			
	***R*^2^**	***p***	Variable *	**Reg. coef. ‡**	***R*^2^**	***p***
Occupied monitors	0.616	0.0032	Temp.	0.026	0.116	0.117
			Moist.	−0.030	0.501	0.002
Discovery rate	0.623	0.0029	Temp.	0.0003	0.119	0.144
			Moist.	−0.0003	0.504	0.002
Leaving rate	0.371	0.0622	Temp.	−0.023	0.088	0.686
			Moist.	0.021	0.283	0.072

Bolded values were statistically significant (*p* < 0.05). * Temp.: Soil temperature, Moist.: Soil moisture. ‡ Reg. coef.: Regression coefficient.
